# Morphological variations and adhesive distribution: a cross-species examination in *Colletotrichum* conidia

**DOI:** 10.3389/ffunb.2024.1481865

**Published:** 2024-12-13

**Authors:** Caleb Oliver Bedsole, Mary Cowser, Timothy Martin, Jillian Hamilton, Lucia Gonzalez Rodriguez, Thomas M. Chappell, Brian D. Shaw

**Affiliations:** Department of Plant Pathology and Microbiology, Texas A&M University, College Station, TX, United States

**Keywords:** *Colletotrichum*, anthracnose, adhesive, development, spore dispersal, conidia, actin

## Abstract

*Colletotrichum* is a globally significant genus of plant pathogens known for causing anthracnose across a diverse array of hosts. Notably, *Colletotrichum graminicola* is a pathogen affecting maize. Annually, the global economic impact of this pathogen reaches billions of US dollars. *C. graminicola* produces conidia that have a characteristic falcate shape and are dispersed by rain. Upon attachment to maize leaves, these conidia develop melanized appressoria to penetrate the leaf surface to initiate disease. Recent findings have emphasized the existence of an adhesive strip on only one side of *C. graminicola* conidia. This strip colocalizes with an actin array, playing a crucial role in facilitating attachment and germination. This asymmetrical adhesive was postulated to enhance spore dispersal by assuring that some conidia do not attach to their initial deposition site. The extent of this asymmetric adhesive phenotype in other *Colletotrichum* species remains unknown, raising questions about its conservation within the genus. This study reveals the ubiquitous presence of an asymmetric adhesive on the conidia across nine isolates of *Colletotrichum*, representing eight species. Morphological differences in conidium shape and adhesive distribution were observed. Significantly, *Colletotrichum truncatum* is unique from other observed species by exhibiting an adhesive strip on both sides of its conidium. Furthermore, in *C. graminicola*, we noted a simultaneous development of the actin array and detachment from its mother cell after spore development. We posit that the study of other *Colletotrichum* members holds promise in elucidating the evolutionary trajectory of this phenotype. Furthermore, these insights may prove instrumental in understanding spore dispersal dynamics across diverse hosts, shedding light on the intricate web of host specificity within the genus.

## 1 Introduction

*Colletotrichum* is a widespread genus of pathogenic fungi with 257 currently accepted species and 15 species complexes (Talhinhas and Baroncelli, 2021). *Colletotrichum* causes devastating diseases on a wide range of plant hosts around the world. *Colletotrichum* species are hemibiotrophic pathogens that initially infect the plant through a biotrophic phase and later switch to a necrotrophic phase (Sukno et al., 2008). Host range varies with species, including numerous economically significant hosts, such as fruits, including apples, mangos, and citrus, as well as grains, such as maize and sorghum. Some species including *C. graminicola, C. incanum*, and *C. sublineola* exhibit a more limited host range, infecting only two hosts, and *C. circinans* infecting five hosts. In contrast, species such as *C. truncatum, C. theobrominicola*, *C. siamense*, and *C. gloeosporioides* have broader host ranges (Talhinhas and Baroncelli, 2021).

One species of note is *Colletotrichum graminicola*, which infects maize and is the causal agent of anthracnose leaf blight and stalk rot. Anthracnose causes a decrease in yield by lowering the photosynthetic ability of the plant; but stalk rot, which multiple pathogens can cause, can cause decay in the plant’s stalk, causing it to be weakened and fall over, making it challenging to harvest. Annually, *C. graminicola* causes billions of US dollars of damage and is one of the world’s most economically relevant fungal plant pathogens (National Agricultural Statistics Service, 2022; Dean et al., 2012; Mueller et al., 2020). Falcate conidia of *C. graminicola* are the primary inoculum and are dispersed by rain (Nordzieke et al., 2019). When the conidia germinate, they form a melanized appressoria, which will penetrate the plant leaf. After penetrating the plant cell, the fungus initiates a brief biotrophic phase, evading the host defense response and transitioning to a necrotrophic phase (Mims and Vaillancourt, 2002; Chaky et al., 2001). During this necrotrophic phase, the fungus induces cell death, invades neighboring host cells, and undergoes further growth (Bergstrom and Nicholson, 1999).

*Colletotrichum* species exhibit notable diversity in their conidia morphologies. For instance, some species, such as *C. graminicola*, display falcate-shaped primary conidia, while others, like *C. gloeosporioides*, produce cylindrical conidia. Intriguingly, some species such as *C. graminicola* showcases an additional morphological variation, generating oval conidia believed to be involved in dispersal within the host plant (Panaccione et al., 1989).

The ecological implications of these distinct conidial morphologies remain poorly understood. Conidial morphology plays a pivotal role in deposition processes and significantly influences the dynamics of spore dispersal, ultimately determining the locations where spores may be distributed (Calhim et al., 2018). A comprehensive understanding of these morphological variations could unveil ecological advantages associated with specific conidial forms, shedding light on the adaptive strategies employed by *Colletotrichum* species in their interactions with host plants.

**Figure 1:**
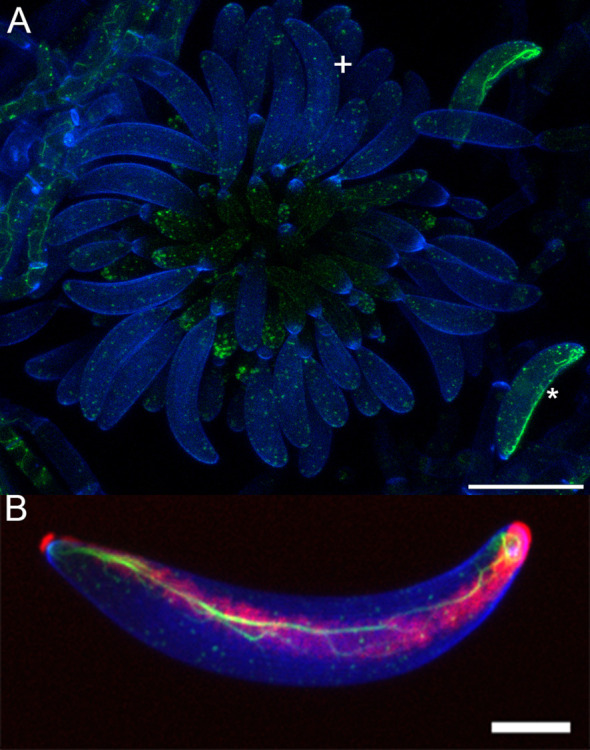
Actin dynamics in *Colletotrichum graminicola* acervulus. **(A)** Stained acervulus from *Colletotrichum graminicola* using 50 µg/mL calcofluor-white cell wall stain (blue) and Lifeact eGFP (Actin) in green. Primary falcate conidia are shown after release, featuring actin cables (*), while conidia not yet fully formed and released exhibit actin patches with no visible actin cables (+). **(B)** Conidium stained with Calcofluor-white cell wall stain (blue), Lifeact eGFP for actin (green), and Concanavalin A for adhesive (red) Demonstrating the colocalization of the adhesive and actin array. Both are Z-stack maximum projections. Scale bar **(A)** = 20 µm, **(B)** = 5 µm.

The Conidial Coin Toss describes the adhesion mechanism of falcate conidia in *Colletotrichum graminicola*, where roughly 50% of conidia attach because of a single-sided adhesive strip, allowing for differential adhesion upon contact (Vasselli et al., 2022). The researchers discovered the adhesion of falcate conidia is crucial for triggering germination and the development of appressoria (Vasselli et al., 2022). It was observed that the adhesion process is rapid and polarized, with a single-sided strip of adhesive material running the length of a single-side of the conidium (**Figure 1**). The adhesive being only present on one side of the conidium caused roughly 50% of conidia to attach which they refer to as the conidial coin toss model. The study also revealed the presence of dynamic actin cables that are polarized to a single face and run from tip to tip. The single-sided adhesive colocalizes with the actin cables which could mean that the actin may play a role in the formation of the adhesive strip. The exact timing of the development of these actin cables is unknown but actin cables had not formed while attached to the acervulus (Vasselli et al., 2022). Furthermore, the research demonstrated that the orientation of conidia upon contact with the substrate determines whether they will rapidly adhere, and those which do not initially adhere can be induced to do so by applying force to reorient the conidia. Without attachment, the spore will not germinate and cannot infect its host (Chaky et al., 2001). Vasselli predicts that this could allow *C. graminicola* to increase its infection court. Vasselli also predicts that the actin array in the falcate conidia could play an essential role in the secretion and or distribution of the adhesive strip.

An alternative hypothesis posits that the asymmetry introduced by the adhesive may increase the likelihood of conidia landing on maize leaves but attaching to the stem, positioned away from UV light, thus potentially slowing down the desiccation process. Furthermore, this mechanism could facilitate conidia landing on lower leaves, where sunlight exposure also is reduced, and potentially in more humid conditions. This differential landing and attachment strategy might confer advantages in terms of moisture retention and less UV radiation, thereby enhancing the conidia’s ability to establish and thrive in environments characterized by varying levels of sunlight and humidity (Estrada et al., 2000; Costa et al., 2021; Braga et al., 2015).

Adhesion has been well characterized in other species of fungi (Vasselli and Shaw, 2022; Apoga and Jansson, 2000; Braun and Howard, 1994). For instance, *Magnaporthe oryzae* spores contain a preformed adhesive at their apex, which is released upon hydration, facilitating adhesion at their tips (Hamer et al., 1988). Similarly *Phyllosticta ampelicida* pycnidiospores also feature preformed adhesives, manifesting in the form of an adhesive sheath and a mucilaginous coating (Shaw and Hoch, 1999; Kuo and Hoch, 1995; Shaw et al., 1998). Calcium ions potentially serve as a triggering factor for conidial germination, mirroring their role in germination processes observed in various other fungal species (Shaw and Hoch, 2001, 1999; Roy et al., 2020).

Characterization of the formation of the actin array could be vital to understanding the developmental timing of the adhesive strip. Although not yet tested, it is reasonable to hypothesize that the actin array serves as a predictive determinant for the deposition site of the adhesive. Vasselli also showed that freshly harvested conidia possess the actin array and adhesive strip (Vasselli et al., 2022). Prior investigation has demonstrated the pivotal role of the actin cytoskeleton in conidiogenesis, exocytosis, and endocytosis in other fungal species, such as *Magnaporthe oryzae*, leading to detrimental effects if inhibited (Gabriel et al., 2006; Li et al., 2020; Qi et al., 2016).

The recent identification of an asymmetric adhesive array in *C. graminicola* prompts an exploration into the prevalence of this unique phenotype across various *Colletotrichum* species. In light of the diverse characteristics exhibited by *Colletotrichum* species, including variations in host range and spore morphology, it becomes imperative to identify which species exhibit the trait of asymmetric adhesive distribution. This specificity is crucial for unraveling the nuanced implications on the attachment and dispersal mechanisms of these distinct species. Past research has proposed that the allantoid (falcate) shape of conidia, as seen in *C. graminicola*, may confer distinct advantages in terms of deposition on above-ground substrates and spore settlement (Webster and Davey, 1984; Calhim et al., 2018). Diversity in host range, spore morphology, and other factors could influence the distribution of the adhesive; observing this in different species could prove vital to our understanding of this initial stage of infection.

## 2 Materials and methods

### 2.1 Media and slide preparation

All cultures were grown on half strength Potato Dextrose Agar (19.5 g PDA/Liter adjusted to 1.5% agar; IBI Scientific, USA) with continuous light and replated weekly. After 10-14 days spores were collected by removing a small square of agar 3cm from the leading edge of mycelia and pipetting 0.5 mL ddH_2_O to suspend the conidia. The concentration was then adjusted to 5x10^5^ for all experiments. To observe conidiogenesis, the culture was grown on tap water agar with 2% agar and three autoclaved rice grains were placed on the surface of the agar to observe single conidiophores. After 14 days of growth with continuous light of cool white fluorescent 1050 Lumens at room temperature, agar blocks were cut into roughly 2 cm squares approximately 3 cm from the edge of the plate and inverted on a coverslip with 30 µL of water (Hickey and Read, 2009). To maintain hydration of the colony, 20 µL of water was added to the top-facing (opposite face of the agar block from the specimen) side of the agar block every hour of imaging, and 150 µL of water was placed in Microcentrifuge Tube Caps from Fisher Scientific and was kept in proximity to the agar block to avoid desiccation.

### 2.2 Microscopy

Microscopic observations were made using an Olympus FV3000 laser scanning confocal system interfaced with an Olympus IX83 inverted microscope with a Galvanometer scanner and High Sensitivity GaAsP PMT detectors. UAPON 100x OTIRF objective (NA = 1.49) with Olympus oil immersion type-F. The motorized stage enables optical sectioning in the z-axis, and the z-drift compensator allows stable time-lapse images over long time periods. Calcofluor-White (Sigma, USA) was excited at 405 nm and the detection wavelength was captured between 430-470 nm. Concanavalin A (Sigma, USA) was excited at 561 nm and the detection wavelength was captured between 570-670 nm. Images were exported via Olympus CellSens software desktop version 2.3, and images were analyzed with the image software ImageJ version 2.14.0 (Schneider et al., 2012) images were prepared in Photoshop version 25.3.1 20231212.r.241 (Adobe Inc.., San Jose, CA, United States, 2019).

### 2.3 Strains and dyes used

A *C. graminicola* strain expressing Lifeact::GFP (CgAB221-17) was used to visualize actin (Wang and Shaw, 2016), all other strains are in **Table 1**. Calcofluor-White (Sigma, USA) was used to visualize the cell wall at a working concentration of 50 µg/mL from a stock solution of 1 mg/mL. Concanavalin A (Sigma, USA) was used to visualize the adhesive at a final working concentration of 200 µg/mL from a stock concentration of 5 mg/mL. Dyes were then added to the spore solutions to allow for visualization of the cell wall and adhesive. Concanavalin A, a lectin that binds glycoproteins, was chosen to label adhesive due to its strong affinity for carbohydrate-rich secretions typical of fungal adhesins. Lifeact::GFP was used to label the actin array, providing a fluorescent marker for actin dynamics that aligns with adhesive strip formation.

**Table 1:** *Colletotrichum* species and strains causing anthracnose represented in this study.

Species	Species Complex	Source	Host	Conidia Shape	BLAST Result Accession Numbers and Identity %
*Colletotrichum sublineola*	Graminicola	Brazos County, Texas	Sorghum (*sorghum bicolor*)	Falcate	ITS: NR_111191.1 99.82% Identity *act*: JQ005834.1 100% Identity
*Colletotrichum graminicola* M1.001	Graminicola	Missouri	Maize (*Zea mays*)	Falcate	ITS: EFQ25829 100% Identity *act*: JQ005830.1 98.82% Identity
*Colletotrichum graminicola* CgAB221-17	Graminicola	Missouri	Maize (*Zea mays*)	Falcate	ITS: EFQ25829 100% Identity *act*: JQ005830.1 98.82% Identity
*Colletotrichum circinans*	Dematium	Zavala County, Texas	Spinach (*Spinacia oleracea*)	Falcate	ITS: MH81329.1 99.65% Identity *act*: GU227953.1 96.96% Identity
*Colletotrichum incanum*	Spaethianum	Gaines County, Texas	Peanut (*Arachis hypogaea*)	Falcate	ITS: NR_160812.1 99.81% Identity *act*: KC110823.1 100% Identity
*Colletotrichum truncatum* (UvTX1)	Truncatum	Uvalde County, Texas	Cotton (*Gossypium* sp.)	Falcate	ITS: NR_144789.1 99.83% Identity *act*: GU227960.1 99.60% Identity
*Colletotrichum truncatum* (UvTX2)	Truncatum	Uvalde County, Texas	Sesame (*Sesamum* sp.)	Falcate	ITS: NR_144789.1 99.65% Identity *act*: GU227960.1 99.20% Identity
*Colletotrichum theobrominicola*	Gloeosporioides	Tarrant County, Texas	Boxwood (*Buxus* sp.*)*	Cylindrical	ITS: OQ716687.1 99.66% Identity *act*: JX009444.1 100% Identity
*Colletotrichum gloeosporioides*	Gloeosporioides	Uvalde County, Texas	Sesame	Cylindrical	ITS: JX010152.1 99.66% Identity *act*: JX009531.1 100% Identity
*Colletotrichum siamense*	Gloeosporioides	Atascosa County, Texas	Strawberry (*Fragaria* × *ananassa*)	Cylindrical	ITS: JX010171.1 99.83% Identity *act*: FJ907423.1 99.59% Identity

### 2.4 DNA extraction, PCR amplification, and sequencing

DNA was extracted from cultures after one week of growth. Once the agar with fungal mycelium was cut out of the plate, it was mixed with lysis buffer (0.5M NaCl, 10mM Tris-HCl pH 7.5, 10mM EDTA, 1% SDS), placed in a bead beater, and followed by the phenol-chloroform method as done previously (Sambrook and Russell, 2006; Wang et al., 2016). After the internal transcribed spacer (ITS) region of 18S rDNA was amplified with PCR using universal primers ITS 4 (TCCTCCGCTTATTGATATGC) and ITS 5 (TCCGTAGGTGAACCTGCGG), as well as for actin (*act*) using act-512F (ATGTGCAAGGCCGGTTTCGC) and act-783R (TACGAGTCCTTCTGGCCCAT). PCR reactions were done with the following settings: Initiation for 5 min at 95°C, denaturation for one minute at 95°C, annealing for 30s at 55°C for ITS and 48°C for *act*, extension for 1 min at 72°C. The Thermal Cycler was run for 35 cycles and then a final extension cycle was run at 72°C for 5 min. Sequencing performed by Eton Bioscience Inc. Sequence quality was determined using SeqTrace software (Stucky, 2012).

### 2.5 Constructing the phylogeny

Once the ITS and *act* region were amplified and sequenced, it was aligned in MEGA 11 using the ClustalW algorithm and manually analyzed and adjusted. Sequences were compared via Standard Nucleotide BLAST (Altschul et al., 1990) comparing to GenBank (Benson et al., 2018). Evolutionary analyses were conducted in MEGA11 (Tamura et al., 2021). Maximum likelihood phylogenetic trees were visualized in FigTree v. 1.4.4 (Rambaut, 2018) and edited in Photoshop version 25.3.1 20231212.r.241 (Adobe Inc., San Jose, CA, United States, 2019) for trait mapping. The resulting phylogeny was compared to (Talhinhas and Baroncelli, 2021).

### 2.6 Surface area calculations

The proportion of cell surface with adhesive coverage was quantified for 50 cells from each of eight species of *Colletotrichum* shown in **Table 1** with 2 biological replicates. From these species there are three conidia morphologies that were observed: falcate with single-sided adhesive, falcate with double sided adhesive, and cylindrical with double sided adhesive. The surface area was computed using the 3D Objects Counter plugin in ImageJ. To facilitate cross-species comparisons, both cell walls and adhesives were considered, with their respective surface areas calculated. The process involved isolating the Calcofluor-White channel (cell wall stain) from the Concanavalin A channel (adhesive), applying the plugin, and determining a threshold to eliminate noise.

### 2.7 Statistical analysis

The proportions of cell surface occupied by adhesive were transformed with a logit transformation. Pairwise means comparisons of adhesive content for were calculated for *Colletotrichum* species and for spore shape morphologies using Tukey’s Range Test. Statistical testing was done using the GLM procedure of SAS v9.4 (SAS Institute Inc. Cary, NC).

## 3 Results

### 3.1 Observations and timing of actin array formation

**Figure 2:**
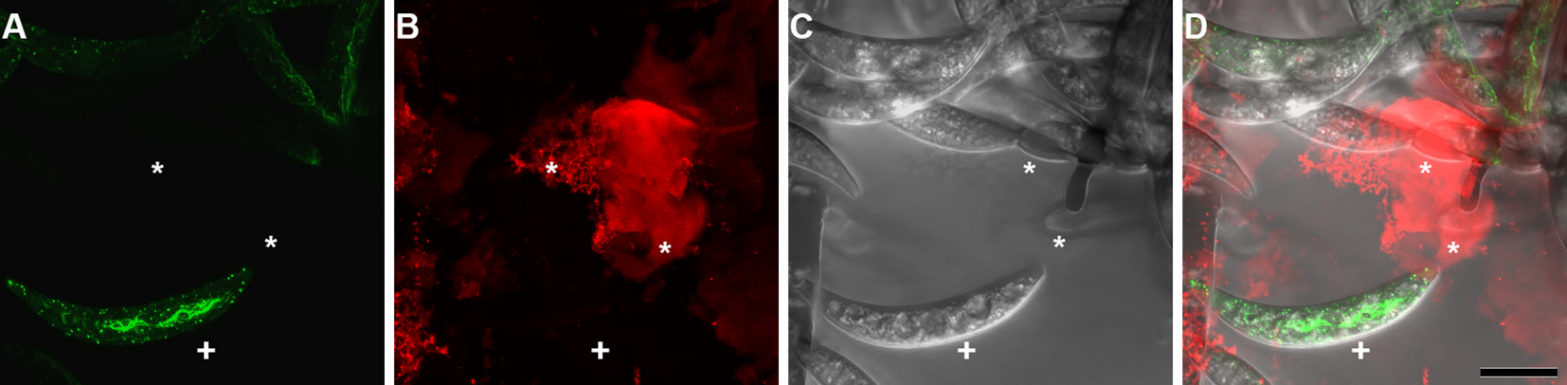
Concanavalin a binding to *Colletotrichum graminicola* Phialide-Associated Mucilage. **(A)** shows Lifeact eGFP (Actin), **(B)** shows Concanavalin A adhesive stain, **(C)** shows the transmitted light, **(D)** shows the overlay, (*) denotes conidiogenous cell mucilage, and (+) denotes unattached conidium with actin array but no observable adhesive. Scale bar = 10 µm.

In order to document the development of the adhesive strip, we used Concanavalin A to label developing conidia, as shown for mature conidia in **Figure 1**. Concanavalin A, which binds to glycoproteins, labeled a distinct mucilage secretion at the cell-cell junction found at each individual phialide and its nascent conidium (**Figure 2**). However, specific binding to the conidial adhesive in acervuli was not observed, likely due to interference from the surrounding mucilage matrix. Therefore, we decided to use the formation of the actin array as a proxy for the formation of the adhesive strip, given the established colocalization between the actin array and adhesive in mature conidia (Vasselli et al., 2022). Therefore, we reasoned that the formation of the actin array would function as an indicator of when the conidium formed the adhesive.

**Figure 3:**
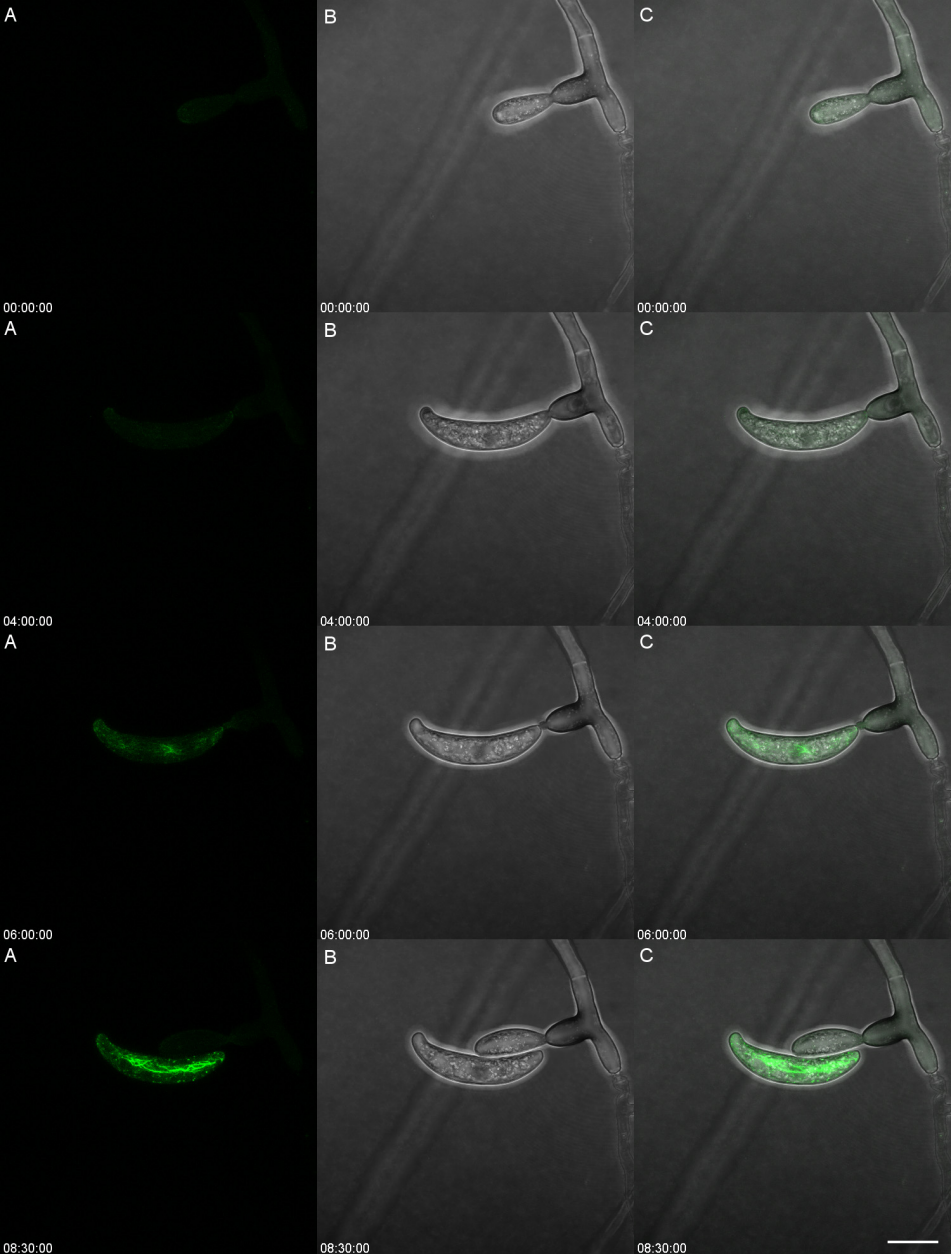
Conidium ontogeny showing the developmental stage at which the actin array Is formed in *Colletotrichum graminicola*. The actin array appears only after spore separation. The left panel **(A)** shows Lifeact eGFP (Actin), the center **(B)** shows transmitted light, and the right **(C)** is the overlay. Time is shown as HH: MM : SS from a timelapse image sequence in a maximum Z stack projection. Video version of Figure 3 is found in **Supplementary Video 1**. Scale bar = 10 µm.

To determine the timing of the actin array development the acervulus was examined using the Lifeact strain of *Colletotrichum graminicola* (CgAB221-17) (Wang and Shaw, 2016). Conidia that remained attached to their mother cell exhibited no discernible actin array; however, they did retain observable actin patches (**Figure 3**) (**Supplementary Video 1**). To determine if actin cables form prior to detachment from the colony, it was necessary to observe nascent conidia emerging from their conidiogenous phialide.

Observations of developing conidia utilizing transmitted light are inhibited by the mucilage secreted around the acervulus that was previously described (Moraes, 1980; Sugui et al., 1998). However, the limitation was overcome with the use of a fluorescent cell wall stain, Calcofluor-White. A total of 4199 conidia were observed that remained attached to their conidiogenous cells. Of these, 99.8% of conidia which remained attached to their conidiogenous cell did not possess any observable actin array or actin cables. In contrast, 1544 conidia that had detached from their conidiogenous cell were observed. Of these, 95.7% of conidia that were detached from their conidiogenous cell displayed a fully formed actin array that ran from tip to tip of the falcate conidium.

This observation led to the hypothesis that the actin array formed only after detachment from the conidiogenous cell. Actin inhibition pharmacological agents were considered but deemed impractical due to the vital roles of the cytoskeleton in conidiogenesis, endocytosis, exocytosis, and hyphal growth (Schultzhaus and Shaw, 2015) (Gabriel et al., 2006; Samaj et al., 2004). However, long-term time-lapsed imaging of a single conidiogenous cell proved useful in determining the timing of the development of the actin array (**Figure 3**). During the initial stages of development of the conidium, there was no observable actin array (00:00:00). Actin patches were observed in the apex of the conidium as the cell developed. When the conidium reached its entire length and appeared fully formed, the actin array was not detectable (04:00:00). The appearance of the actin array occurs only after the conidium detaches from its conidiogenous cell (06:00:00). Within three more hours, the actin array becomes fully formed and reaches its maximum intensity (08:30:00). The production of the next blastic conidium aids in the detachment of the previous conidium.

These observations support the conclusion that the actin array is formed after detachment of the newly formed conidium. Here, we use the presence of the actin array as a proxy for the formation of the adhesive strip, as previously described by the colocalization of the adhesive and the actin array (Vasselli et al., 2022). The presence of large amounts of gelatinous secretion around all acervuli of *C. graminicola* (Mondal and Parbery, 2005; Sugui et al., 1998) precludes a similar analysis of the development of the conidium adhesive because Concanavalin A also reacts with this matrix (**Figure 2**), making observation of the development of the adhesive strip impossible using Concanavalin A.

### 3.2 Molecular characterization and comparative phylogeny of *Colletotrichum* isolates

**Figure 4:**
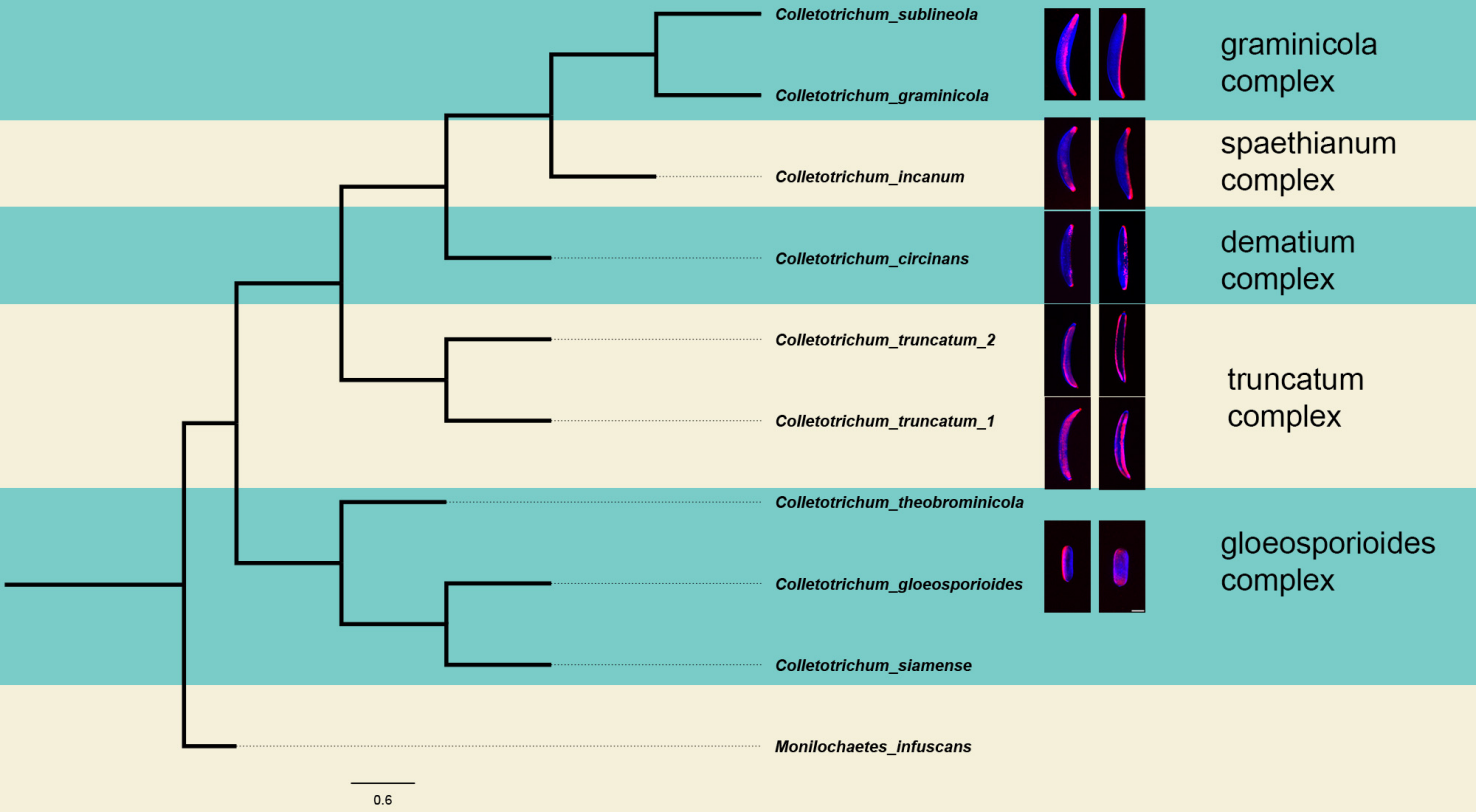
Phylogeny of *Colletotrichum*. The phylogeny was reconstructed from a sequence alignment of the ITS region between primers its4 and its5, and the *act* region between the primers act-512F and act-783R. This phylogenetic tree illustrates the distinctions among the species under investigation in this study. The tree was constructed using the Maximum Likelihood method and the Tamura-Nei model with a bootstrap value of 1000. The phylogenetic were visualized in FigTree v. 1.4.4. The topology of this tree agrees with the placement of each species in their respective sections of the genus previously published (Talhinhas and Baroncelli, 2021).

Comparison of sequence between Internal Transcribe Region (ITS4/ITS5), as well as the actin gene (act-512F/act-783R) for each of the isolates, revealed a sequence match that was 96.96-100% identity to accessions in GenBank (**Table 1**). An important observation is that in the BLAST analysis, the top results for *C. truncatum (UvTX1)* and *C. truncatum (UvTX2)* resulted in a 99.65% and 99.83% identity, respectively, with their ITS sequences to *C. jasminigenum*, a species synonymized with *C. truncatum* according to (Talhinhas and Baroncelli, 2021). Additionally, the *act* sequences of *C. truncatum (UvTX1)* and *C. truncatum (UvTX2)* results had a 99.6% and 99.2% identity, respectively, with *C. truncatum.* Each of the isolates belonged to five distinct species complexes of *Colletotrichum* as described by (Talhinhas and Baroncelli, 2021). These include the graminicola complex, the dematium complex, the spaethianum complex, the truncatum complex, and the gloeosporioides complex. To best represent the distribution of spore morphology and adhesive distribution, a maximum likelihood phylogenetic tree was generated (**Figure 4**). The topology of the tree matched that described by (Talhinhas and Baroncelli, 2021). In constructing the phylogeny presented in **Figure 4**, this study employs a more limited dataset, encompassing only eight species, and a narrower set of genes, in comparison to a comprehensive work conducted by (Talhinhas and Baroncelli, 2021). This phylogenetic tree, though narrower in scope, offers a supplementary perspective to the existing literature.

### 3.3 Asymmetric adhesive distribution is broadly distributed in the genus *Colletotrichum* adhesive distribution

**Figure 5:**
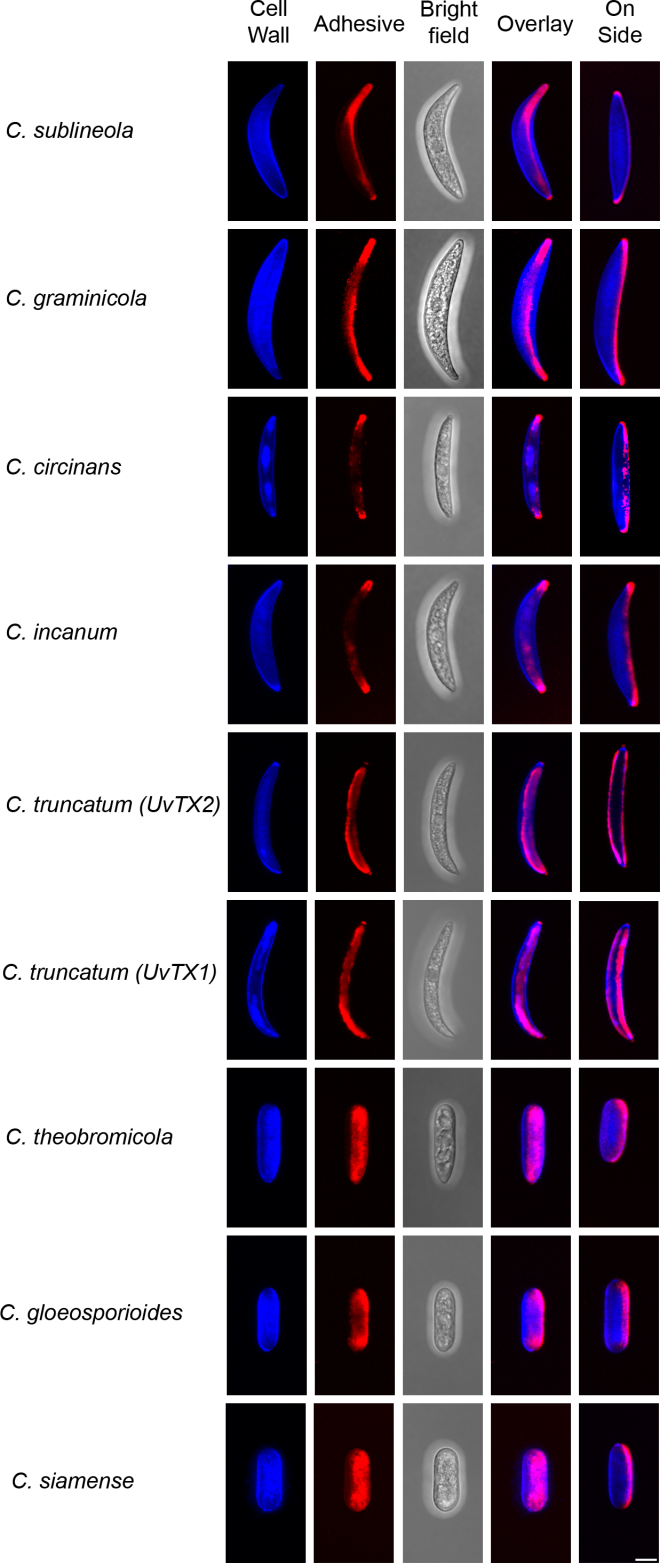
Adhesive distribution and conidia morphology. Various species of *Colletotrichum* from the phylogeny tree (**Figure 4**) are shown both as attached and on the side of conidia, presented in a maximum Z-stack projection. The cell wall was stained using Calcofluor-White (blue), while the adhesive was stained with Concanavalin A (red). All images were captured using consistent settings and procedures. Each image is representative of observations from 50 spores analyzed per species. Scale bar = 5 µm.

The observed species manifest variations in conidia morphology and adhesive distribution (**Figure 5**). A consistent observation across all species, with the exception of *C. truncatum*, is the presence of an asymmetric adhesive distribution. Remarkably, *C. truncatum* deviates from this pattern by exhibiting adhesives on both sides of the conidia, despite its overall similarity in morphology to other falcate-shaped conidia members. Species such as *C. theobrominicola*, *C. gloeosporioides*, and *C. siamense*, display cylindrical-shaped conidia. Despite this disparity in shape, these spores exhibit an asymmetrical adhesive, aligning with the prevalent trend observed in most *Colletotrichum* species. In **Figure 5**, this adhesive asymmetry is visible in the ‘on side’ view of the spores, where the adhesive appears on only one side of the conidium, despite seeming more evenly distributed in the overlay image due to the adhesive’s contact with the coverslip. These findings suggest that adhesive asymmetry in cylindrical and falcate spores may be an adaptive feature facilitating targeted attachment and improved dispersal across varying host surfaces. Such asymmetry could enhance spore adhesion efficiency, positioning *Colletotrichum* species for successful colonization in diverse environments.

**Figure 6:**
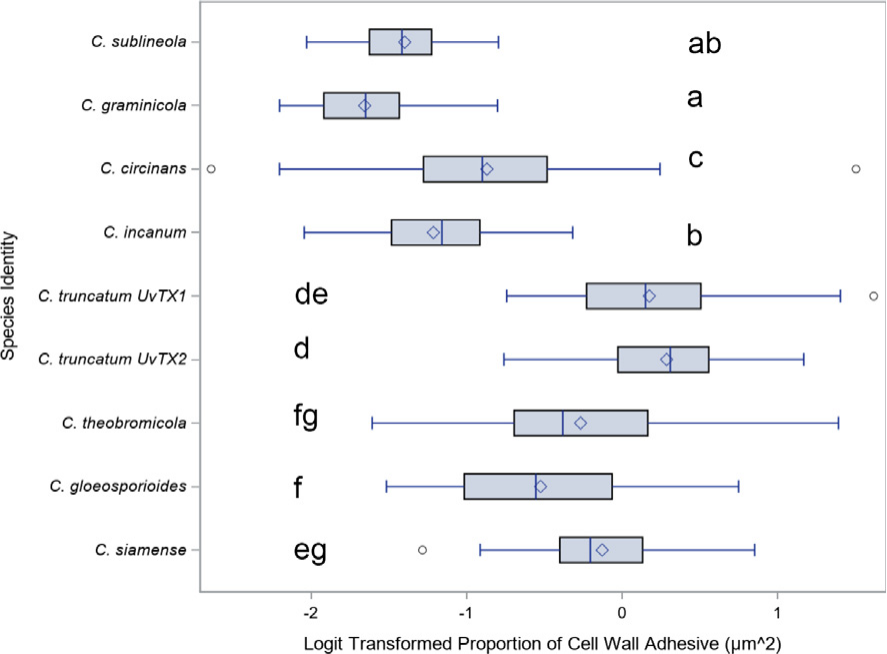
Mean and range comparisons of the ratio of adhesive area to spore surface area for *Colletotrichum* species. Box and whisker plot for the Logit transformation of the proportion of cell wall area covered by adhesive, delineated by species. Highlighting the differences in adhesive coverage on the cell wall among different species. Lettering denotes species that are significantly different as calculated using Tukey’s range test.

**Figure 7:**
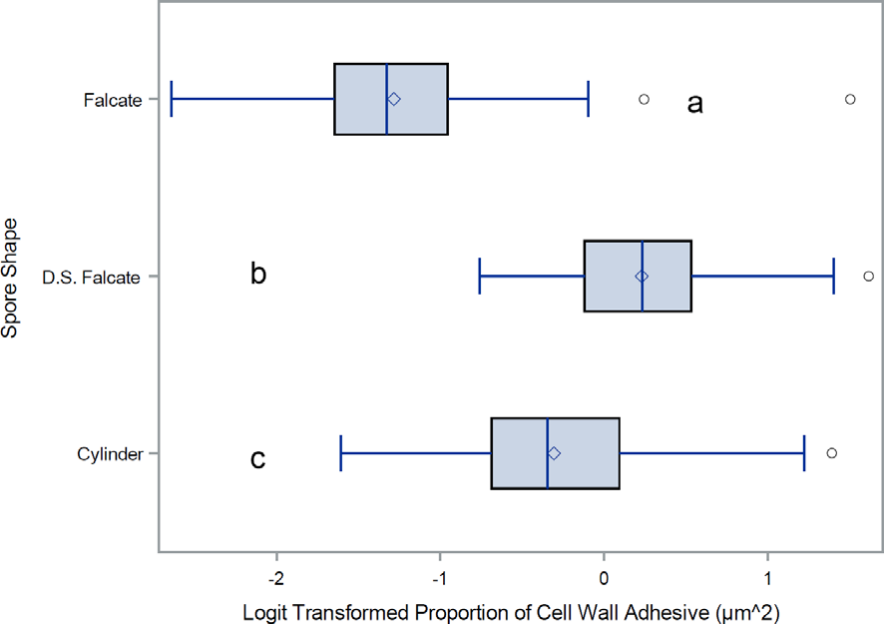
Mean and range comparisons of adhesive ratios for *Colletotrichum* spore shapes. Box and whisker plot for the Logit transformation of the proportion of cell wall covered by adhesive, delineated by spore morphology. D.S. Falcate indicates falcate shaped conidia with adhesive deposition on both sides of the spore. Lettering denotes species that are significantly different as calculated using Tukey’s range test.

For a quantitative analysis of the variations in conidia adhesion, the surface area of the adhesive was calculated (**Figure 6**). Species with falcate conidia, *C. sublineola*, *C. graminicola, C. circinans*, and *C. incanum* as a group, significantly differ from other spore morphologies in proportional adhesive surface area (**Figure 7**). However, within the falcate group, species exhibit variability in adhesive proportions, as shown by distinct statistical groupings (**Figure 6**). Similarly, *C. theobrominicola, C. gloeosporioides*, and *C. siamense* share the cylindrical conidia morphology and, as a group, significantly differ from other spore morphologies in proportional adhesive surface area (**Figure 7**). Within the cylindrical group, species exhibit distinct statistical groupings, reflecting variability in adhesive proportions (**Figure 6**). *C. truncatum* represents a unique conidia morphology among the species tested, because although it is falcate in shape, it possesses adhesive strip on both sides of the spore. The two strains of *C. truncatum*, *C. truncatum* (UvTX1) and *C. truncatum* (UvTX2), which were isolated from different host plants, were not significantly different from each other in adhesive surface area proportion. However, these strains still formed distinct groupings compared to most other species. When modeled as a function of spore shape, the proportion of adhesive surface significantly differed between all conidia morphologies (p<0.05) (**Figure 7**).

The lack of significant difference in adhesive surface area between the two strains of *C. truncatum* suggests that adhesive properties may not vary significantly within a species, even when strains originate from different hosts. However, the grouping of *C. truncatum* strains with other species suggests that broader morphological or ecological factors may also influence adhesive surface area proportions. Further studies could investigate the interplay between species-specific traits and environmental factors to better understand the drivers of adhesive variation within and across *Colletotrichum* species.

## 4 Discussion

### 4.1 Actin array formation in conidia

The observed dynamics in actin array formation and adhesive distribution across various *Colletotrichum* species present intriguing insights into the intricate processes governing conidial development and attachment. This investigation into the formation of the actin array during conidial development uncovered distinct patterns. Conidia detached from their conidiogenous cells consistently displayed an observable asymmetric actin array. Notably, incipient conidia still attached to their phialidic cell did not exhibit any observable actin array. This consistent pattern, observed in 99.8% of conidia while connected to their conidiogenous cell, suggests a crucial link between actin array formation and the detachment phase. Furthermore, detached conidia displayed the actin array in 95.7% of cases. One reason that some conidia do not possess the actin array when detached could be that some are forcibly detached as a consequence of mounting the slide, causing the cell to die before it could close the septum of the new conidium. The development of the actin array was clarified through time-lapsed imaging, unveiling a sequence of events. During initial development, no observable actin array was detected. Actin patches emerged in the apex as the conidium matured, yet the complete formation of the actin array became apparent only after detachment. This process underscores the speed of a pivotal event in the conidial life cycle. A reasonable assumption would be that the adhesive is secreted after the formation of the co-localized actin array (**Figure 1**). However, the feasibility of directly assessing this hypothesis is impeded by the inherent limitation that the Con A dye, employed for visualizing the adhesive, intricately interacts with the mucilage matrix, rendering precise discrimination unattainable (**Figure 2**).

### 4.2 Phylogeny

In this study, five distinct species complexes within the *Colletotrichum* genus were observed. Despite the existence of fifteen distinct species complexes, our study underscores the pervasive conservation of the asymmetric adhesive in the majority of the observed complexes. The observed variations in both conidial and adhesive morphologies suggest a potential correlation with host-specific differences and diverse leaf morphologies. Future research should investigate not only the adhesive localization within the remaining species complexes of *Colletotrichum* but also within other fungal genera. Such endeavors promise to enrich our understanding of adhesive mechanisms and their evolutionary implications across diverse fungal taxa. Such investigations hold the potential to enhance our understanding of adhesive mechanisms and their evolutionary implications.

### 4.3 Adhesive distribution

Conidia morphology in the genus *Colletotrichum* can be generally described by two morphological traits: spore shape, and adhesive deposition. *Colletotrichum* conidia can be either falcate or cylindrical in shape, with an intriguing discovery of asymmetric adhesive distribution unique to this genus. While the precise implications remain uncertain, there is speculation that this phenomenon may contribute to enhanced spore dispersal. Remarkably, *C. truncatum* conidia are falcate shaped, but the adhesive is deposited on both sides of the cell. These morphological differences between species present potential lines of inquiry host range modeling as a function of conidial adhesive distribution and shape. Further research should explore adhesive localization in additional species complexes within *Colletotrichum*. While the precise interplay between the asymmetric adhesive and its consequential impact on spore distribution remains an ongoing investigation, the influence of conidia shape on spore distribution emerges as another intriguing aspect for exploration. The elucidation of these dynamics not only underscores the complexity of adhesion but also calls for a comprehensive understanding of their ecological implications. This correlation prompts contemplation on the potential adaptive significance of adhesive abundance in *Colletotrichum* species, which could thereby enrich our understanding of host-pathogen interactions within this genus. The morphological diversity and adhesive variations observed across *Colletotrichum* species may reflect evolutionary adaptations to different ecological niches and host types. The observed asymmetry in adhesive distribution could be a strategy for selective attachment on surfaces that maximizes spore survival and dispersal efficiency. These morphological traits may be particularly advantageous in environments with variable humidity or exposure, where selective adhesion confers ecological benefits. Further studies investigating environmental factors affecting adhesive patterns could provide valuable insights into the ecological roles of these traits within the genus.

## 5 Conclusion

This study highlights the prevalence of an asymmetric adhesive in various *Colletotrichum* species, including the economically significant *C. graminicola*. We found that most species of *Colletotrichum* observed possess an asymmetric adhesive. We also discovered that *C. truncatum* exhibits a symmetric adhesive, setting it apart from other observed species, as shown by the even distribution visible from both the overlay and side views in **Figure 5**. Our findings shed light on the evolutionary trajectory of this phenotype and its potential role in spore dispersal dynamics across diverse hosts. Further investigation into the development of the actin array and adhesive strip in other *Colletotrichum* species could provide valuable insights into the intricate web of host specificity within the genus. The recent discovery of a symmetric adhesive in *C. truncatum* raises intriguing questions regarding the underlying molecular mechanisms governing adhesive secretion. This newfound characteristic initiates a compelling exploration into its potential implications for the spatial dynamics of the actin array. Drawing on the colocalization between the adhesive distribution and the actin array observed in *C. graminicola*, unraveling the significance of this symmetric adhesive in terms of potential variations in actin array localization and its role in adhesive secretion becomes paramount. Conducting comprehensive comparative analyses offers the potential to reveal distinctive features of *C. truncatum* adhesive biology. This, in turn, can offer valuable insights into the species-specific variations in coordinated processes essential for a successful infection. While *C. truncatum* (UvTX1) and *C. truncatum* (UvTX2) were not significantly different in adhesive surface area proportions when compared directly, they exhibited distinct statistical groupings with other species (**Figure 6**). This suggests that, while adhesive properties may be conserved within the species, broader ecological or morphological factors may influence these patterns. Both strains were found on different crops, and recently, some species have been synonymized with *C. truncatum.* This raises interesting questions about whether adhesive traits reflect conserved features or host-associated adaptations. Future investigations should investigate the adhesive distribution of other *Colletotrichum* species, examining its potential influence on spore dispersal. The influence of adhesive distribution could vary among species with diverse host ranges, necessitating a comprehensive investigation to elucidate its role in the efficient dispersal of various *Colletotrichum* species. Ultimately, our research may prove instrumental in developing more effective strategies for controlling anthracnose and other devastating plant diseases caused by this genus of fungal pathogens.

## Data Availability

The datasets presented in this article are not readily available. Requests to access the datasets should be directed to Brian.Shaw@ag.tamu.edu.

## Supplementary material

Supplementary Video 1 Conidium ontogeny showing the developmental stage at which the actin array is formed in *Colletotrichum graminicola*. The actin array appears only after spore separation. The left panel shows Lifeact eGFP (Actin). The actin array first begins to form around 05:00:00 in the video. The center panel shows transmitted light, and the right panel is the overlay. Time is shown as HH: MM : SS from a timelapse image sequence in a maximum Z stack projection.

Supplementary Video 2 Conidia morphology rotation. Rotation of z-stack, maximum projects of conidia of *Colletotrichum graminicola, C. truncatum, C. gloeosporioides*. Cell wall is with Calcofluor-white (blue) and carbohydrate/glycoprotein is labeled with Concanavalin A (Red). The video demonstrates the differences in conidia morphology with *C. graminicola* as an example of falcate conidia with an asymmetric adhesive, *C. truncatum* with a falcate morphology with an adhesive on two faces of the conidium, and *C. gloeosporioides* with cylindrical conidia with an asymmetric adhesive covering one hemisphere of the conidium. Scale bar = 10 µm.
